# Screening for fetal alcohol spectrum disorder in infants and young children

**DOI:** 10.3389/adar.2023.11125

**Published:** 2023-07-14

**Authors:** Lauren Fleming, Connor Sheridan, Douglas Waite, Marilyn G. Klug, Larry Burd

**Affiliations:** ^1^ School of Medicine and Health Sciences, University of North Dakota, Grand Forks, ND, United States; ^2^ Developmental/Behavioral Pediatrics, BronxCare Health System, Mount Sinai School of Medicine, Bronx, NY, United States; ^3^ Department of Population Health, School of Medicine and Health Sciences, University of North Dakota, Grand Forks, ND, United States; ^4^ Department of Pediatrics, School of Medicine and Health Sciences, University of North Dakota, Grand Forks, ND, United States

**Keywords:** screening, fetal alcohol spectrum disorder, prenatal alcohol exposure, neurodevelopmental disorder, infancy

## Abstract

**Introduction:** With an estimated prevalence of up to five percent in the general population, fetal alcohol spectrum disorders (FASD) are the most common neurodevelopmental disorder and more prevalent than autism. Early identification and subsequent early intervention have the potential to improve developmental trajectory of children with FASD. In addition, new research suggests supplementation with choline may ameliorate the developmental impairments associated with prenatal alcohol exposure. Availability of a screening tool with acceptable epidemiologic performance criteria may be clinical useful in identification of young children at increased risk for FASD. In this paper we describe the Early Fetal Alcohol Spectrum Disorder Screening Test (E-FAST) to identify young children at increased risk for an FASD.

**Methods:** We developed the E-FAST dataset from previously published studies, comprised of 281 children under 5 years of age, 180 (64.1%) were diagnosed with FASD and 101 (35.9%) were non-FASD.

**Analysis:** The analysis identified seven useful variables (prenatal alcohol exposure, ADHD (Attention Deficit Hyperactivity Disorder), foster care or adopted, small OFC (occipital frontal circumference), communication impairments, impaired social skills, and cognitive deficits. All variables were categorized as yes/no for ease of use in a screening tool. Risk ratios for each of the seven indicators were estimated using two-way table analyses. Weights for each variable were estimated based on the relative strength of their odds ratios.

**Results:** The average age was 2.7 years of age (S.D. 1.29) and ranged from infant (6.4%) to 4 years old (35.9%). Maternal alcohol use alone had a sensitivity of 0.97, specificity 0.65, and accuracy 0.86. For the combined seven variables, sensitivity was 0.94, specificity 0.74, and accuracy 0.87. Thus, the seven-item E-FAST screen had acceptable epidemiologic screening characteristics.

**Discussion:** In the United States, up to 547 infants with FASD are born each day which far exceeds the capacity of multidisciplinary diagnostic clinics. During routine clinical management of infants and young children the use of an evidence-based screening tool provides a time efficient means to exclude large numbers of young children from further follow-up for FASD. Conversely, a positive screen identifies a smaller number of children at increased risk for FASD requiring more intensive evaluation and follow-up.

## Introduction

Despite widespread public information efforts to increase awareness of the risks of alcohol use during pregnancy, rates of alcohol use in pregnant women have been increasing [1, 2]. A CDC study found that 13.5% of pregnant women reported current alcohol use and 5.2% reported drinking four or more drinks on an occasion(binge-drinking) [3]. Among women of child-bearing age, 53.6% used alcohol in the past month and 18.2% binge drank [4]. Because up to 50% of pregnancies are unplanned, many of these women will have children with prenatal alcohol exposure [5]. Upon confirmation of pregnancy, most women quit or reduce alcohol use but 10.2% continue to drink [4] and recent studies indicate that over 8% of pregnant women are drinking at the end of pregnancy [6].

Prenatal alcohol exposure has been demonstrated to have a negative effect on the developmental trajectories of infants and young children language, cognitive and motor skills [7, 8]. In addition prenatal alcohol exposure increase risk for fetal alcohol spectrum disorder (FASD). FASD is the most common cause of noninheritable developmental disability in the United States. A study of first-graders in four regions of the United States found a conservatively estimated prevalence rate of 1.1%–5.0% [9]. Global prevalence of FASD is at least 2.2% with wide variation across countries and subpopulations [10]. These prevalence rates demonstrate that FASD is a disorder as common as any other medical condition physicians diagnose and treat each day. Yet both screening for prenatal alcohol exposure or for FASD is far from routine.

Even when prenatal exposure to other substances such as cannabis, cocaine, or opioids is documented in newborn medical records, screening for prenatal alcohol exposure is notably absent in most obstetric, pediatric, and child welfare records. In addition to stigma against women who use alcohol during pregnancy [11], identification of children with an FASD is complicated by the need to obtain a history of prenatal alcohol exposure. Accurate ascertainment of history of prenatal alcohol exposure is complicated by the frequency in which children at the highest risk for an FASD often do not reside with their biological parents. In a recent study of 151 children/young adults screened for FASD, only 4 (2.6%) were raised by biological family members. Information on prenatal care and alcohol and other substance exposure can be very challenging to obtain in these circumstances [12].

Another challenge in the diagnosis of FASD is the lack of a screening tool for practitioners to utilize to easily identify infants and young children at risk for an FASD. A comprehensive review of FASD screening tools identified 20 unique screening tools for FASD utilized in 45 cross-sectional or case-controlled studies [13]. Typical screening tools analyze facial dysmorphology, growth retardation, behavioral and developmental indicators, along with characteristics of parents. Of the 20 screening tools, only 5 studies included children under the age of six and only 3 included children ages 2 to 3; no studies included children under 2. Another recent study retrospectively analyzed 151 subjects all of which were seen in a national FASD clinic [12]. The ages in this study ranged from 3.75 to 22 years. Within this range, 78% (118 of 151) were between ages 6 and 16 and no data was presented for children under age 3.7 years. These two articles demonstrate the need for an early screening for FASD to allow early identification and intervention to maximize child neurodevelopment. In this manuscript we discuss a new screening tool for FASD which was developed for use in young children during routine healthcare visits or when developmental delays are a concern.

In this manuscript we respond to this issue by reporting on the development of a screening test for FASD for very young children (Early Fetal Alcohol Spectrum Disorder Screen for Young Children) the E-FAST.

## Methods

The E-FAST data set was developed from three deidentified data sets which have been previously published (FAS Diagnostic Checklist 2002; *n* = 405, FAS Screen 1997; *n* = 264, and data from the ARND (alcohol related neurodevelopmental disorder) Behavioral Checklist *n* = 47. The initial criteria for diagnosis is determination of prenatal alcohol exposure. The diagnostic criteria and the methodology for diagnosis has been presented in detail in [14]. In brief, each child with a diagnosis of FASD had an exposure assessment which consisted of the One-Question Screen “When was your last drink?”, and a Maternal Risk Score and a dosimetry assessment [14–16]. This data is then reviewed and a five item Likert scale is used to assess clinician confidence in exposure. The five scale intervals are confirmed prenatal alcohol exposure, prenatal alcohol exposure, no-reporter, no exposure and confirmed no-prenatal alcohol exposure. The dosimetry assessment collects data on drinking days per week, drinks per drinking day, number of binge episodes (four or more standard drinks on an occasion), what is a drink(s) and days per week for cigarette smoking and number of cigarettes per smoking day. The second criterion includes meeting the neurobehavioral phenotype for FASD [14, 17]. This included assessment of relevant records for previous intellectual testing, neuropsychological testing, adaptive behavior testing, assessment for attention deficit hyperactivity disorder, speech and language testing, memory testing, executive function testing, vision and hearing testing, number of adverse childhood experiences, and number of foster home placements. Where needed additional testing was completed during the assessment or by referral. Children with other comorbid disorders were not excluded from the FASD group if they met criteria for FASD.

The ARND data set (*n* = 47) did not include data on occipital frontal head circumference (OFC) or the size of the child (height or weight). Those values were imputed using estimated values from a logistic regression that used smoking, age, sex, and race as input variables.

### Determination of screening variables

The final dataset included 281 children under age 5 years included in the analysis. All variables were categorized as yes/no for ease of use as variables in a screening tool. Occipital frontal circumference (OFCs) under the 20th percentile for their age were considered at risk. Children placed in foster care or adopted were combined into one variable. The variable ADHD included a diagnosis of ADHD and behavioral observations of attention deficits or impulsiveness. Speech and language disorders included diagnosed speech and language disorders, stuttering, or observations of communication impairments. The variable social problems included social skills deficits, difficulty or inability to make friends, or noticeable deficits in relating to other children. Cognitive impairments included IQ below 85, learning disability, memory impairments, need for special education services, therapy, or early intervention services due to learning deficits.

Multiple other variables were available but not included in the analysis. They included maternal variables such as age and smoking status, information on the father or siblings (substance use, diagnoses, mortality), and variables regarding the child such as other diagnosis or birth information. These were excluded since the variables did not have sufficient observations or positive values to be useable. Other variables such as mother’s age, were so closely related to other variables their collinearity rendered them unusable. Birthweight, which had to be controlled for gestation, was too complicated and OFC gave an easier and quick measure of a child’s size. There were no facial or other physical features included other than OFC. Other variables like depression were not included since these are typically diagnosed in older children.

## Statistical analysis

The initial analysis of the E-FAST dataset was designed to identify useful screening variables. Risk ratios for each of the potential indicators were estimated using two-way table analyses. Multiple logistic regressions of the variables predicting FASD were estimated for just maternal drinking alone, the other six variables, and all seven variables together. From these regressions, weights for each variable were estimated based on the relative strength of their odds ratios. Composite variables from different combinations of indicator weights were summed and used to produce receiver operating characteristic curves (ROC). Variable combinations included 1) maternal alcohol exposure alone, 2) ADHD, speech and language disorders, social skill deficits, cognitive impairments. These in turn were used to estimate cutoff values for the composite variable models that maximized sensitivity and specificity of the indicators.

## Results

Of the 281 children who had screening variables useable for this analysis, 180 (64.1%) were diagnosed with FASD and 101 (35.9%) were non-FASD. In this sample 161 (57.5%) were male and 84 (29.9%) were white. The average age was 2.67 years (S.D. 1.29) and ranged from infant (6.4%) to 4 years old (35.9%).

**FIGURE 1:**
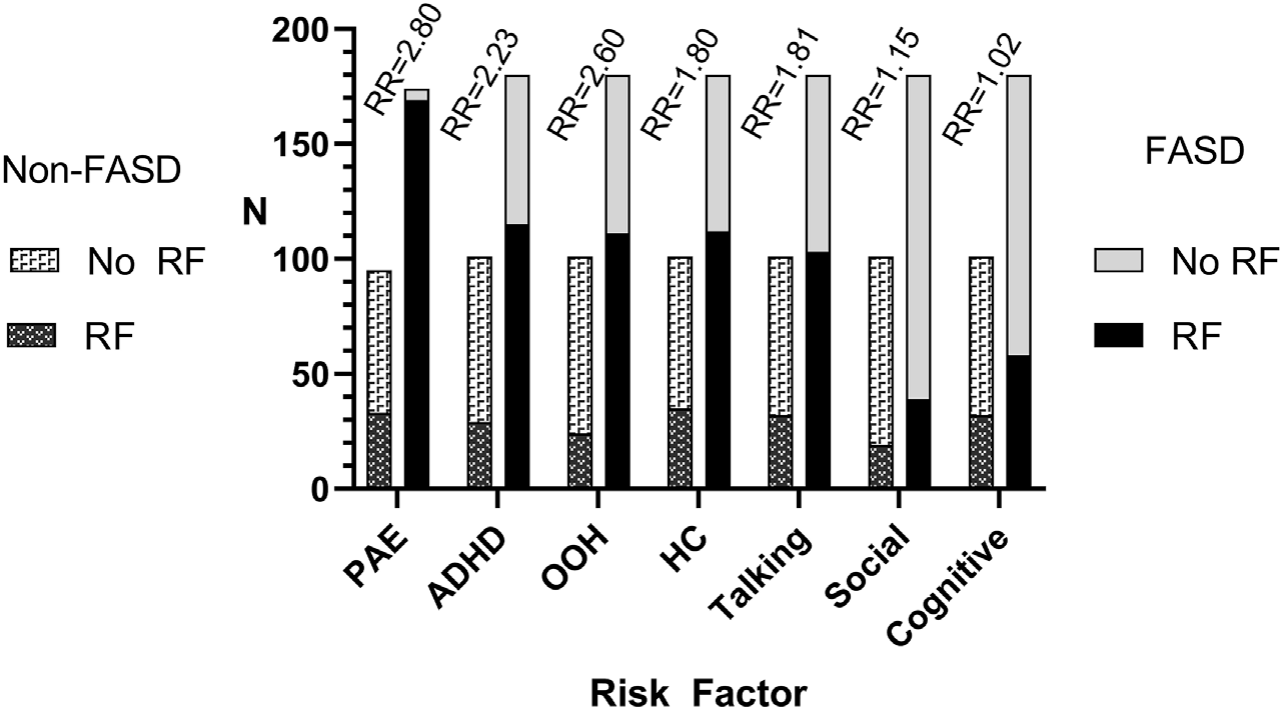
The number of young children with fetal alcohol spectrum disorder (FASD) and non-FASD comparison children who had or did not have one of the seven E-FAST risk factors included in the analysis. PAE, prenatal alcohol exposure; ADHD, attention deficit hyperactivity disorder; OOH, foster care, adopted, living out of parents home; HC, head circumference; Talking, verbal communication impairments; Social, social skill deficits; Cognitive, cognitive impairments.

**FIGURE 2:**
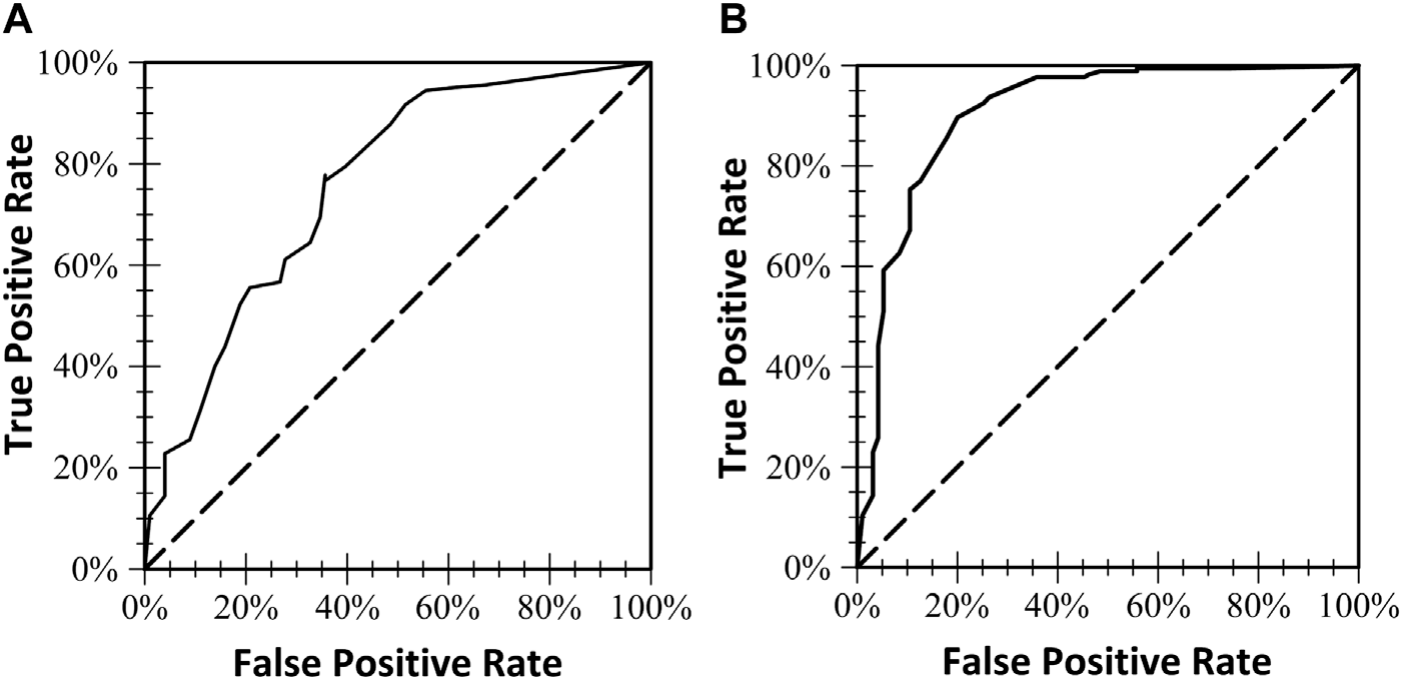
The two receiver operating characteristics (ROC) curves and the area under the curve for each of the two models. **(A)** Included six of the seven E-FAST Screening variables (prenatal alcohol use was not included in this model). **(B)** Models all seven E-FAST screening variables.

The initial analysis of the E-FAST dataset identified seven variables that were useful. Figure 1 presents the number of children with any of the seven screening variables by FASD group (FASD or non-FASD). Alcohol use during pregnancy (variable 1) was reported in nearly all children with a diagnosis of FASD. Children diagnosed with FASD were over twice as likely to have ADHD (variable 2) or be in foster care or adopted (variable 3), and nearly twice as likely to have small OFC (variable 4) or communication impairments (variable 5). Children with FASD were also at increased risk for socialization (variable 6) or cognitive deficits (variable 7). Table 1 shows the logistic regressions of different combinations of E-FAST risk factors. Alcohol use during pregnancy had the highest odds ratio of 63.5. When the six risk factors were taken together, without alcohol use, ADHD had the highest OR of 12.3, Children who were in foster care or adopted OR was 2.9, and small OFC OR was 4.5. Talking, social, and cognitive difficulties were not significant with ORs near one. Weights for composite scores, based on ORs, ranged from 20 points for alcohol to 1 point for cognitive impairments. Figure 2.

**TABLE 1:** Logistic regressions for different combinations of variables for the E-FAST Screen for FASD and the assigned variable weights for the items included in the screen.

N	E-FAST variables	E-FAST variable #	B Est	*P*	OR	Weight
	Model 1					
269	PAE	1	2.176	<.001	63.502	20
	Model 2					
234	ADHD	2	2.5081	<.001	12.282	10
	OOH	3	1.0584	.0044	2.882	3
	HC	4	1.5070	<.001	4.513	5
	Talking	5	0.1725	.6629	1.188	1
	Social	6	0.2975	.7309	1.346	1
	Cognitive	7	−0.0102	.9837	0.990	1
	Model 3					
222	PAE	1	3.2671	<.001	26.236	20
	ADHD	2	2.4848	<.001	11.999	10
	OOH	3	0.9122	.0497	2.490	3
	HC	4	1.3010	.004	3.676	5
	Talking	5	0.1173	.8154	1.124	1
	Cognitive	7	0.1990	.7672	1.220	1

PAE, prenatal alcohol exposure; ADHD, attention deficit hyperactivity disorder; OOH, foster care, adopted, living out of parents home; HC, head circumference; Talking, verbal communication impairments; Social, social skill deficits; Cognitive, cognitive impairments.

ROC curves were used to find cutoff scores for the screening combinations of the weights. Table 2 shows the sensitivity, specificity, accuracy, and area under the ROC curve (AUC) for maternal alcohol use alone, risk factors without alcohol use, and all variables together. Sensitivity of the E-FAST was highest for the two models where alcohol use during pregnancy data was available (.97 and .94), though specificity increased with the addition of the other six risk variables (.74). Although the E-FAST ROC values were lowest for positive screening scores without alcohol use, the six variable model accuracy was still over 70%.

**TABLE 2:** Receiver operating characteristics (ROC) analysis using suggested cutoffs for combinations of screening variables for FASD three variable models of the E-FAST. The E-FAST variables numbers are in parenthesis.

N	Cutoff	Variables	Sensitivity	Specificity	Accuracy	AUC
269	≥20	PAE (1)	.9713	.6526	.8587	.8119
281	≥7	ADHD (2), OOH (3), HC (4), Talking (5), Social (6), and Cognitive (7)	.7778	.6436	.7295	.7579
269	≥21	PAE (1), ADHD (2), OOH (3), HC (4), Talking (5), Social (6), and Cognitive (7)	.9368	.7368	.8662	.9061

PAE, prenatal alcohol exposure; ADHD, attention deficit hyperactivity disorder; OOH, foster care, adopted, living out of parents home; HC, head circumference; Talking, verbal communication impairments; Social, social skill deficits; Cognitive, cognitive impairments.

**FIGURE 3:**
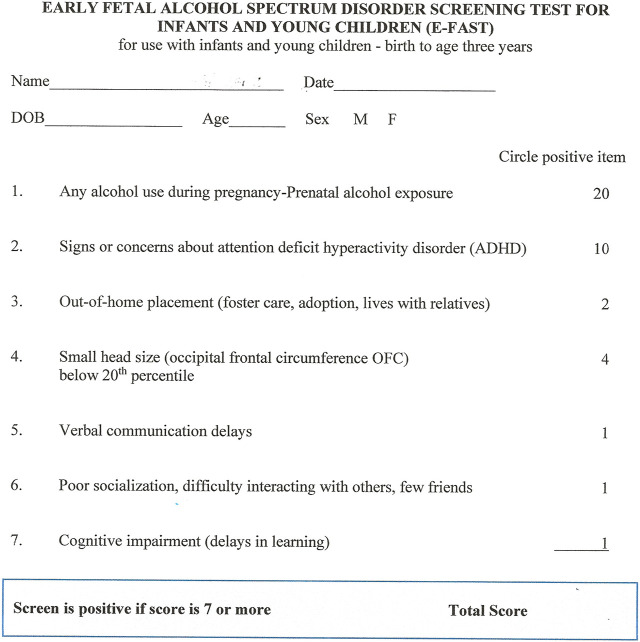
The early fetal alcohol spectrum disorder screening test for infants and young children (E-FAST).

Figure 3 is the final E-FAST Screening tool. Scoring the E-FASD is simple. If the score exceeds 7, the screen is positive and this suggests the child is at increased risk for having and FASD. Among the options for the clinician is placing the child in a more intensive follow-up system to increase the frequency of well child visits. This effort could include increased screening for common problems these would include vision and hearing, sleep, speech and language delays. Identification of exposure to adverse experiences of childhood or placement in foster care would suggest increased risk for FASD. In the accompanying manuscript we describe an office-based approach for identification and management FASD developed for pediatricians and other pediatric providers (8).

## Discussion

In the United States, FASD prevalence rates are as high as one in 20 school-aged children or about 5% of first grade students (^7^)This suggests that in an annual birth cohort of 4.0 million births in the United States approximately 200,000 are infants are born with FASD each year. This figure equates to 3,800 infants with FASD born each week or 547 every day. Only a very small fraction of children with FASD can be seen in the few multidisciplinary clinics currently operating in the United States. Strategies for office-based identification by pediatricians and other early child health providers is urgently needed to facilitate identification and intervention in early childhood [8]. One potentially useful strategy is the E-FAST which can be used to exclude a majority of infants and young children from those who require further assessment for FASD. This is an important function of screening in an office-based practice. The E-FAST functions has acceptable epidemiologic performance characteristics and clinicians could expect that most infants and young children with a negative E-FAST screen will not have FASD.

The second role of screening is to identify a population of infants and young children requiring further assessment. The E-FAST is useful for screening for potential cases since some of the common features of FASD are included as variables in the screen. Lastly, a history of prenatal alcohol exposure may often be a key in the differential diagnosis of FASD. On the E-FAST a positive finding of maternal alcohol use represents a positive screen. However, where information on prenatal alcohol exposure is not available the other six variables can be used to screen. Since information on prenatal alcohol exposure may not always be accurate, the other six variables on the E-FAST can provide a rational for ongoing observation and alternative strategies for exposure assessment.

Concerns about ADHD prior to diagnosis would also provide a useful rational for ongoing monitoring for FASD as well since 50% of children diagnosed with FASD also have either ADHD or concerns about ADHD [18]. FASD is much more prevalent among children in foster care or who have been adopted [19]. The E-FAST can be used in this setting where maternal disclosure of alcohol use has potential for hindering reunification or when direct interview of the mother is not possible.

The primary gatekeepers to identification of children with an FASD are general pediatricians and other front-line early childhood providers. Children identified by the E-FASD screen as at risk for FASD, can be readily referred for further evaluation. Given the paucity of FASD diagnostic centers in the United States especially in rural areas, further assessment might be completed by telemedicine evaluation which has the potential to dramatically increase the number of children diagnosed with an FASD [20]. In the management of children in foster care or with developmental disorders routine screening for exposure to prenatal alcohol is an important step. The basis of FASD diagnosis rests upon prenatal alcohol exposure. The E-FAST-screening tool should not be considered an alternative to screening all children for prenatal alcohol exposure which is a separate clinical issue. The E-FAST offer a rapid means for front-line practitioners to screen, identify, and refer children for FASD diagnosis while also providing a rational for starting intervention services. Finally, new research suggests that pre- and even post-natal choline supplementation may have potential for mitigating the effects of prenatal alcohol exposure [21]. While evidence for the benefits of choline supplementation remains sparse, many experts and families may decide to use choline given the minimal risk of side effects of supplementation in children at risk for FASD.

This study is limited by lack its relatively small sample size, limited inclusion of children from a wide range of diverse settings, cultures and ethnic diversity. When other tools become available for use in this young population comparative studies should promptly be initiated to contrast different approaches to determine optimal screening strategies. It may be that different diagnostic criteria for FASD would result in different screening variables and these variables may have different screening weights in other screening tools.

Further areas of study should also include population-based application of the E-FAST in general pediatric settings to assess the efficiency, efficacy and effectiveness of the tool. Such studies may also refine the performance characteristics of the tool. In addition, further pathways for referral of children screening positive should be clarified to help practitioners know about next steps after a positive screen or diagnosis of FASD [8]). Despite these limitations, this study provides an initial strategy to improve the identification of children with FASD. More importantly, the E-FAST screen has the potential for use to address under identification of children with FASD. Early identification and entry into services is an important management strategy for children and families impacted by FASD. This screening tool may be a part of a system to identify young children who are currently undiagnosed and a first step in entry into appropriate intervention services.

## Data Availability

The raw data supporting the conclusion of this article will be made available by the authors, without undue reservation.
